# Preoperative Immunonutrition vs. Standard Dietary Advice in Normo-Nourished Patients Undergoing Fast-Track Laparoscopic Colorectal Surgery

**DOI:** 10.3390/jcm10030413

**Published:** 2021-01-22

**Authors:** Manfredi Tesauro, Andrea M. Guida, Leandro Siragusa, Bruno Sensi, Vittoria Bellato, Nicola Di Daniele, Andrea Divizia, Marzia Franceschilli, Giuseppe S. Sica

**Affiliations:** 1 Department of Systems Medicine, University Tor Vergata, 00133 Rome, Italy; mtesauro@tiscali.it (M.T.); didaniele@med.uniroma2.it (N.D.D.); 2 Department of Surgical Science, University Tor Vergata, 00133 Rome, Italy; andreamartina.guida@gmail.com (A.M.G.); leandros93@hotmail.it (L.S.); brunosensi@outlook.it (B.S.); vittoria.bellato@gmail.com (V.B.); andreadivizia@live.it (A.D.); marzia.franceschilli@gmail.com (M.F.)

**Keywords:** immunonutrition, ERAS, laparoscopy, colorectal surgery

## Abstract

Immunonutrition (IN) appears to reduce infective complications and in-hospital length of stay (LOS) after major gastrointestinal surgery, but its use in normo-nourished patients is still controversial. The primary aim of this comparative observational study was to evaluate if pre-operative IN reduces in-hospital stay in patients undergoing laparoscopic colorectal resection for cancer under an enhanced recovery after surgery (ERAS) program. The influence of IN on time to first bowel movements, time to full oral diet tolerance, number and type of complications, reasons of prolonged LOS and readmission rate was evaluated as secondary outcome. Patients undergoing ERAS laparoscopic colorectal resection between December 2016 and December 2019 were reviewed. Patients who have received preoperative IN (group A) were compared to those receiving standard dietary advice (group B). Mean in-hospital LOS was significantly shorter in patients receiving preoperative IN than standard dietary advice (4.85 ± 2.25 days vs. 6.06 ± 3.95 days; *p* < 0.0492). No differences in secondary outcomes were observed. Preoperative IN associated with ERAS protocol in normo-nourished patients undergoing laparoscopic colorectal cancer resection seems to reduce LOS.

## 1. Introduction

Immunonutrition (IN) has been introduced in clinical practice to improve nutritional status and positively influence host immune response to surgical stress [1,2,3]. IN requires the use of special immune-modulating nutrients in higher doses than standard nutritional protocols. 

IN is reported to lower infections rate and length of stay (LOS) after major gastrointestinal surgery in general. [4,5]. Furthermore, in most ERAS protocols, a large amount of space is dedicated to perioperative nutrition, carbohydrates load and restriction in parenteral fluids administration [6,7]. 

Few studies focus on patients with colorectal cancer undergoing elective surgery [6] and preoperative IN in normo-nourished patients undergoing enhanced-recovery after surgery (ERAS) laparoscopic colorectal surgery needs a better evaluation due to controversial or low-quality evidence [8,9,10]. 

The primary outcome of this study was to evaluate a protocol of preoperative IN and perioperative maltodextrins load in patients undergoing ERAS laparoscopic colorectal resection for cancer.

## 2. Material and Methods

We undertook a retrospective cohort study following the “strengthening the reporting of observational studies in epidemiology (STROBE)” statement [11]. Consecutive patients undergoing elective ERAS laparoscopic colorectal resections for cancer between December 2016 and December 2019 at Minimally Invasive Surgery Unit of Tor Vergata University Hospital were identified from a prospectively maintained database, recording continuous and discrete variables regarding biometric data, patient-related risk factors, type of surgical procedure, compliance to ERAS items (including preoperative IN) and outcomes. Nutritional status was evaluated using the Mini Nutritional Assessment-Short Form (MNA^®^-SF), a 6-questions based malnutrition screening test [12]. Patients were considered normo-nourished in case of MNA-SF score ≥ 12. 

During the postoperative period, any complication (intended as any adverse event during the follow-up period) including infective complications, anastomotic leak (AL, regardless of its clinical significance), surgical site infections (SSI, defined according to the Centre for Disease Control and Prevention, CDC/NHNS) [13], pneumonia (clinical symptoms, confirmed by radiological examination) and ileus was recorded and graded according to the Clavien–Dindo classification [14]. 

### 2.1. Inclusion Criteria and Subdivision in Groups

We included all adult patients over 18 years of age, normo-nourish and diagnosed with primary colorectal carcinoma undergoing elective laparoscopic colorectal resection for cancer following a protocol of enhanced recovery in a single unit over 3 years (Table 1).

Patients were divided in two groups depending on the different pre-operative nutritional protocol. In group A, we have put patients who have accepted to receive 5 days of pre-operative IN and maltodextrins load a few hours before surgery; in group B, we have put patients who have followed dietary advice only (3 days of high protein and low fiber diet and clear fluids with sugar and simple carbohydrates up to four hours before the operation).

### 2.2. Exclusion Criteria 

The exclusion criteria included: age below 18 years, malnutrition, inflammatory bowel disease, low rectal cancer patients who had neo-adjuvant therapy, acquired or congenital immunodeficiency, preoperative infection, American Society Anesthesiology (ASA) IV, pregnancy, surgery in emergency, conversion to open surgery and multivisceral resections.

Inclusion and exclusion criteria are summarized in Table 2.

### 2.3. Endpoints

The primary outcome of this study was to evaluate differences amongst the two groups in the overall postoperative length of stay (LOS), defined as the number of postoperative days (POD) of hospitalization. Secondary endpoints were: time to postoperative food intake (defined as tolerance to solid diet), time to first defecation, 30-days postoperative complications, surgical site infections, anastomotic leak, pneumonia, ileus, readmission rate, mortality and prolonged length of stay (PLOS), defined as any LOS greater than 1.5-times the median LOS in the final matched cohort. All the endpoints were analyzed in both group, A and B.

### 2.4. Statistical Analysis

All quantitative data were expressed as mean ± standard deviation (SD), while categorical data were expressed with percentage frequencies. Univariate analysis for both primary and secondary outcomes were performed with Student’s t-test for nonparametric data; two-tailed Chi-square or Fisher tests were used to compare differences in frequencies (SPSS, Inc., Chicago, IL, USA). Results were considered as statistically significant when *p* < 0.05.

### 2.5. Ethics

This study was conducted according to the international ethical recommendations on clinical research established by the Helsinki Declaration [16]. All patients included were provided written informed consent. The study was registered at ClinicalTrials.gov (Preoperative immunonutrition vs. standard dietary advice in normo-nourished patients undergoing fast-track laparoscopic colorectal surgery; NCT04692545). 

## 3. Results

### 3.1. Study Population

**Figure 1:**
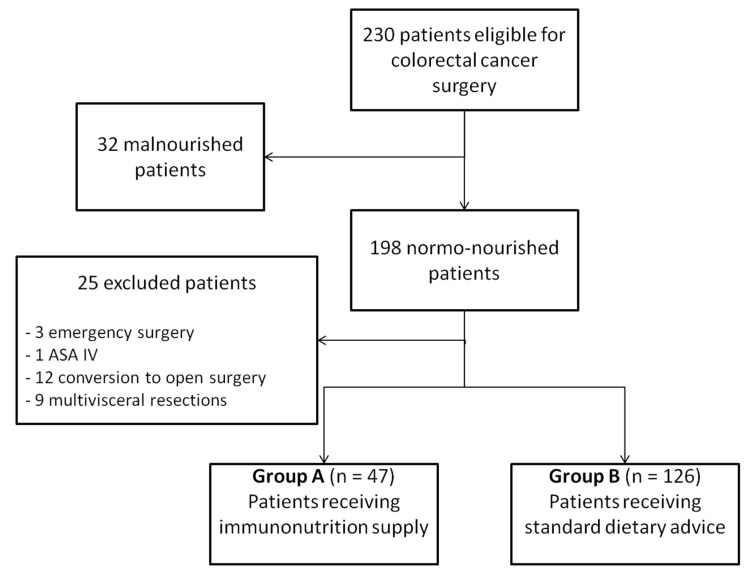
Patients’ selection.

From December 2016 to December 2019, 230 consecutive patients underwent colorectal resection for cancer in a single unit. Fifty-seven patients (24.8%) did not meet the inclusion criteria and were excluded from the analysis: thirty-two (13.9%) were found malnourished, three patients had emergency surgery, one was classified at high risk for surgery (ASA IV), twelve laparoscopic operations were eventually converted to open surgery and nine patients had multivisceral resections. One-hundred seventy-three patients were subsequently included for the study purpose, of which 47 (27%) had preoperative IN with Impact oral^®^ (Nestlé Health Science S.A.) and maltodextrins (group A), while 126 (73%) had decided on following standard dietary advices (group B) (Figure 1).

Baselines patients’ characteristics are summarized in Table 3. The two groups were comparable with respect to age, sex, BMI, comorbidities, ASA score, nutritional status assessed using the MNA-SF, preoperative albumin and type of surgical procedures. No differences in the groups were recorded in compliance to the ERAS items in use at our institution during the study period (Table 4).

### 3.2. Outcomes

Primary and secondary outcomes are shown in Table 5.

**Figure 2:**
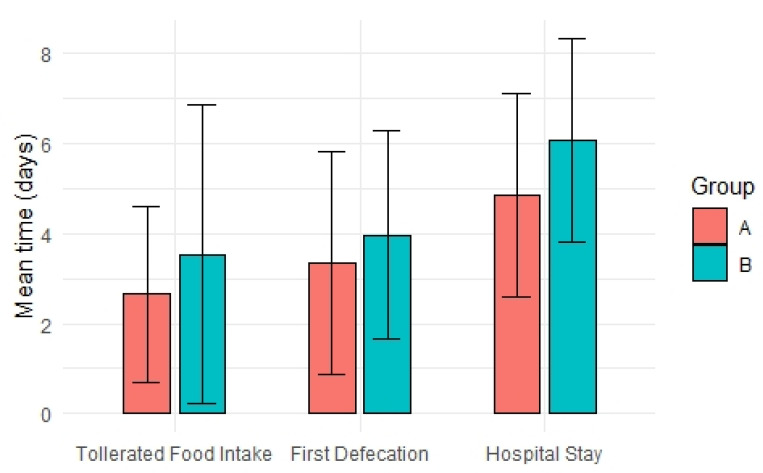
Tolerated food intake, first defecation and Length of Stay (LOS) comparison between groups.

Mean and median LOS for group A and B were found to be significantly different: 4.85 ± 2.25 vs. 6.06 ± 3.95 (*p* < 0.0492) (95% CI −2.44–0.02) and 4 vs. 6 days, respectively (*p* < 0.0466). There were no differences in time to first defecation (A: 3.33 ± 2.47 vs. B: 3.96 ± 2.31) (95% CI −1.42–0.16) nor in time to solid diet tolerance (A: 2.65 ± 1.94 vs. B: 3.54 ± 3.31) (95% CI −1.9–0.12) (Figure 2).

No differences in major postoperative complications, PLOS (any LOS greater than 8 days), readmission rate, reoperation (group A: 2 leaks, 2 small bowel occlusions; group B: 6 leaks, 1 bleeding, 3 small bowel occlusions, 1 negative exploration) and SSI rate were found between groups. One patient in Group B died on day 2 of massive bowel ischemia and was subsequently excluded from the analysis of LOS and functional outcomes. No mortality was observed in Group A.

## 4. Discussion

In most protocols of enhanced-recovery after surgery (ERAS), large space is dedicated to perioperative nutrition, carbohydrates load and restriction in parenteral fluids administration [8,9,10]. It is interesting to note that despite obesity being a known risk factor for several medical condition such as diabetes hypertension and also colon cancer, up to 50% of patients receiving treatment for colorectal carcinoma seem to be malnourished [17,18]. The rationale for the use of preoperative IN with leptin and arginine is to be found in the postoperative catabolic state. The proinflammatory tendency and dysfunctional immune response may lead to increased infections rate and a prolonged wound and anastomosis healing process [19,20,21,22,23,24,25].

Leptin stimulates both endothelin-1 and nitric oxide activity [26,27]. Arginine is a key element in wound and anastomosis healing, being the only precursor of nitric oxide and proline, involved respectively in innate immune response and collagen deposition [19,20,21,22,28,29]. RNA supply is necessary to avoid T-lymphocyte decrease and to raise IL-2 levels and CD4/CD8 ratio [19,20,21,22,30,31], whereas omega-3 and -6 play an anti-inflammatory role by contrasting oxidative injury, downregulating arachidonic acid and generating resolvins [28,32]. The addition of preoperative complex carbohydrates loading prevents the temporary insulin-resistance due to cortisol and glucagon raised after surgery, which is an independent factor increasing LOS [33,34,35].

Strong evidence supports preoperative IN in malnourished patients undergoing major abdominal surgery (defined as any operation creating any gastrointestinal anastomosis or involving parenchymal resection of the liver or pancreas) [5,36,37,38]. Preoperative IN is currently recommended in all major surgery candidates by French guidelines [39], in all cancer patients by ASPEN guidelines [40] and only in malnourished patients undergoing major cancer surgery by ESPEN [41,42]. However, its role in normo-nourished patients is still a matter of debate and the large amount of evidence supporting preoperative IN has significant bias (reporting, publication and industry). Nevertheless, preoperative IN is strongly recommended based on moderate grade evidence by the 2018 ERAS Society Guidelines [28,42]. Our study focuses on preoperative IN in a homogeneous group of normo-nourished colorectal cancer patients undergoing ERAS laparoscopic surgery. The proposed preoperative IN was Impact^®^ oral, a nutritional drink enriched with electrolytes, vitamins and immuno-nutrients as n3 n6 fatty acid, arginine and RNA in a 236 mL brick to be taken 3 times a day for 5 days, before surgery (total Kcal = 5.1). All patients were provided with a leaflet regarding potential advantages of IN and an explicatory note on carbohydrates load. In fact, patients who agreed on IN (group A) were also given two spoons of maltodextrins diluted in 400 mL of water, early the day of the operation. However, over 70% of patients choose not to take preoperative IN (group B); thus, they were asked to follow a fiber-poor and protein-rich diet for 3 days and to drink clear fluids, possibly enriched with sugar and electrolytes. No records on the compliance on the prescribed diet are available, but almost all accepted tea and rusks or rice waffles with jam or honey up to 4 h before surgery.

To assess patients’ nutritional status, we used the MNA-SF questionnaire, a 6-questions based malnutrition screening test designed by Nestlé, capable to spot not only malnourished patients but also patients at high-risk of malnutrition [12].

We found a significantly lower LOS in the group of patients who had preoperative IN when compared to the control group, despite the lack of evidence of a synergic effect on post-operative complications rate, including infective complications and leak rate. Our results are in favor of preoperative IN in colorectal cancer surgery in a cohort of patients undergoing ERAS laparoscopic surgery. Several studies evaluated IN on surgical outcomes, including LOS, in heterogeneous cohorts of patients, such as patients undergoing different major abdominal surgical procedures or patients with various gastrointestinal cancers [43,44,45,46]. In 2017, a metanalysis on IN was published in BJS, finding a significant reduction in LOS of −1.79 days after major abdominal surgery. However, most of the included papers suffered from evident risks of bias. In fact, only 35 out of 83 trials reported precise random sequence with low risks of bias and half of the trials did not report blinding measures nor defined prospective endpoints [5]. An even more substantial reduction in LOS was found in the group undergoing hepato-pancreatic-biliary (HPB) surgery (mean reduction −2.39). However, HPB surgery and in general every major open abdominal surgery causes exceptional stress to the body, with complications and mortality rates up to 45% and 3–7%, respectively [38]. On the contrary, laparoscopic surgery and fast-track protocols seems to reduce surgical stress with noticeable advantages on recovery, LOS, blood loss and complications [44,47].

Advantages in perioperative IN can be found also in the metanalysis published in 2019 by Zhang et al. However, not only is this collection focused on gastrointestinal cancer surgery in general, but also the greatest LOS reduction is found in the postoperative supplementation group (mean reduction −1.95) [4]. Furthermore, the analysis of the subgroup containing only colorectal cancer patients pointed out less noticeable findings.

Only a few authors have focused their analysis on normo-nourished colorectal cancer patients. In particular, the Spanish RCT by Moya et al. compares IN versus standard high calories nutrition in patients undergoing colorectal surgery within an ERAS protocol, demonstrating no statistically significant difference in terms of LOS [48]. The overall ERAS compliance in this trial was about 80%, which is higher than the mean Spanish adherence rate (64%) reported by the POWER study [49]. Both laparoscopic and open surgeries were included in the study, without performing a multivariate analysis to rule out differences; it is, therefore, impossible to evaluate the effect of IN in the group ERAS + laparoscopy, which is the only association that seems to reduce surgical stress and potentially lower LOS [44].

Finally, also in the metanalysis by Xu et al., which found a mean LOS reduction of 2.35 days, it is difficult to draw straight conclusions because of the heterogeneity of the studies. Furthermore, they included small sample sizes series and series with benign diseases, with different surgical approaches, postoperative care and type of nutritional drinks [6].

In our single unit study, we included only patients with colon and rectal cancer who received preoperative IN followed by ERAS laparoscopic surgery.

Major limitations are intrinsic to the observational design of our study, with the potential for residual bias and confounders from measured and unmeasured variables.

However, patients who received IN were not selected, but they agreed on the proposed protocol and accepted to buy the nutritional drinks, not dispensed by our regional health system. Clearly, another possible bias could be that of a selection based on social status that would select a group with a higher standard of life compared to a less wealthy one, but neither BMI nor preoperative albumin (considered as nutritional parameters), nor nutritional status analyzed with the MNA-SF screening test, were different amongst groups.

Another limitation of the study regards the sample exiguity: the statistical significance of the primary outcome (LOS) was near the statistical cut-off and a greater sample might have not confirmed these results. Furthermore, the primary outcome was the LOS and not when the patient was “fit to be discharged”; some confounding factors could have influenced the results. Finally, the limited number of cases could have impaired the possibility to highlight differences in secondary endpoints.

However, this observational study has a valuable hypothesis—a better post-operative outcome in patients receiving preoperative IN in a homogeneous cohort of patients—that has generated a clinical investigation in a single surgical unit, with high degree of uniformity in the procedural path. Further research, with a larger sample may be justified giving the lack of well-conducted trials.

## 5. Conclusions

Results from this observational study suggest that preoperative IN may reduce postoperative in-hospital LOS in patients with cancer of the colon and rectum undergoing ERAS laparoscopic colorectal resection.

## Figures and Tables

**Table 1:** Enhance Recovery After Surgery items and adherence criteria following 2013 guidelines [15].

Items	Adherence Criteria	
Preoperative	Information, education and counseling	➢ Patients receive detailed information regarding perioperative program before surgical procedure in order to reduce anxiety
	Optimization	➢ Smoking and alcohol consumption are stopped four weeks before surgery ➢ Physical activity before surgery is encouraged
	Bowel preparation	➢ Changes according to local and international guidelines
	Oral carbohydrates load and preoperative fasting	➢ Carbohydrates rich beverage is given preoperatively ➢ Diabetic patients receive carbohydrates load along with diabetic medications ➢ Preoperative fasting is limited to 2 h for clear fluids ➢ Preoperative fasting is limited to 6 h for milk and solid food
	Preanesthetic medication	➢ No long- or medium-action sedatives are routinely administered ➢ Short-acting sedatives can be given to ease the administration of spinal, epidural or loco-regional anesthesia
	Antithrombotic prophylaxis	➢ Patients are provided with graduate compression stockings ➢ Patients receive pharmacological prophylaxis with low molecular weight heparin (LMWH) ➢ Pharmacological prophylaxis is administered for 28 days after surgery in case of malignancy
	Antibiotic prophylaxis and skin preparation	➢ Patients are administered IV antibiotic 30–60 min before surgery ➢ Skin preparation is obtained with chlorhexidine-alcohol
Intraoperative	Standard anesthetic protocol	➢ General anesthesia is performed with short-acting anesthetics to allow rapid awakening ➢ To reduce the metabolic stress response, fluid therapy, analgesia and hemodynamic changes are monitored ➢ Opioids are avoided unless necessary
	PONV prophylaxis	➢ Postoperative nausea/vomiting (PONV) prophylaxis is administered in all patients with ≥2 risk factors (Apfel score) through a multimodal approach ➢ PONV treatment is obtained with a multimodal approach
	Minimally invasive surgery	➢ Patients undergone laparoscopic surgery (including conversions to open surgery on an intention-to-treat basis)
	Prevention of hypothermia	➢ Intraoperative body temperature is maintained >36 °C with warmed fluids and/or thermic blankets ➢ Hyperpyrexia is avoided carefully monitoring intraoperative body temperature
	Intraoperative fluid management	➢ Restrictive fluid therapy (defined as maintenance fluids at <2 mL/kg/h) or goal-oriented fluid therapy (stroke volume) is adopted ➢ Intravenous fluids are discontinued as soon as possible
	Multimodal analgesia	➢ More than two drugs or analgesia strategies (TAP-block or spinal anesthesia for minimally invasive surgery; thoracic epidural anesthesia for open surgery) are administered in order to reduce the use of opiates
	No nasogastric tube	➢ No nasogastric tube policy
	No drain	➢ As general policy, no drains. However, if placed, would be removed the following day.
Postoperative	Bladder catheter	➢ Urinary catheter usually placed for right colectomy and always for left colectomy and anterior resections. Removed on postoperative day (POD) 1; up to POD 2 in case of rectal dissection
	Gut motility stimulation	➢ Patients receive chewing-gum twice daily starting on POD 1
	Early mobilization	➢ Patients are passively mobilized on POD 0 ➢ Patients are actively mobilized on POD 1
	Early oral feeding	➢ Patients receive liquid oral diet as soon as they recover from anesthesia and then semisolid diet
	Pre-discharge check	➢ Patients are checked just before discharge ➢ Adequate oral intake, bowel function, pain control, active mobilization, no clinical/serological evidence of any postoperative complication, full agreement to go home

**Table 2:** Inclusion and exclusion criteria.

**Inclusion criteria**	Age > 18 years Patients diagnosed with primary colorectal carcinoma Patients who underwent elective laparoscopic colorectal resection following a protocol of enhanced recovery after surgery Normo-nourished patients (MNA-SF score ≥ 12) ASA I, II, III
**Exclusion criteria**	Age < 18 years Malnutrition (MNA-SF score < 12) Inflammatory bowel disease Rectal cancer patients who had neoadiuvant radiotherapy or chemotherapy Acquired or congenital immunodeficiency Preoperative infection Pregnancy Emergency setting Conversion to open surgery Multivisceral resections ASA IV

**Table 3:** Results of preliminary data.

Parameters	Group A (Immunonutrition) (*n* = 47)		Group B (Standard) (*n* = 126)		*P*
Age (mean, SD)	65.63 ± 12.24		66.29 ± 12.3		0.7536
Sex					
Male (%)	28	59.6%	77	61.1%	0.8627
Female (%)	19	40.4%	49	38.9%	
Preoperative BMI (mean, SD)	25.98 ± 3.48		25.6 ± 4.1		0.5736
ASA score					0.7183
1 (%)	9	19.2%	26	20.6%	
2 (%)	27	57.4%	64	50.8%	
3 (%)	11	23.4%	36	28.6%	
Morbidity %					
Diabetes	2	4.3%	16	12.7%	0.1602
Hypertension	15	31.9%	58	46%	0.1195
Heart disease	9	19.2%	28	22.2%	0.8352
Respiratory disease	3	6.4%	11	11.1%	0.7610
Preoperative albumin (gr/dl) (mean, SD)	3.96 ± 0.58		3.91 ± 0.52		0.5865
MNA-SF score	13.2 ± 0.89		12.93 ± 0.83		0.4033
Surgical procedure %					0.1501
Right hemicolectomy	16	34.1%	33	26.2%	
Left hemicolectomy	26	43.8%	59	46.8%	
Anterior rectal resection	4	22.6%	28	22.2%	
Other	1	4.1%	6	4.8%	

**Table 4:** Compliance with ERAS protocol.

Parameters	Group A (Immunonutrition) (*n* = 47)	Group B (Standard) (*n* = 126)	*p*-Value
Perioperative information and counselling	47 (100%)	126 (100%)	1
Preoperative carbohydrates load	47 (100%)	119 (94.4%)	0.1917
No pre-anesthetic medication	41 (87.2%))	105 (83.3%)	0.6411
Antimicrobial prophylaxis and skin preparation	47 (100%)	126 (100%)	1
Thromboprophylaxis	47 (100%)	126 (100%)	1
Goal directed fluids	36 (76.6%)	93 (73.8%)	0.8449
Hypothermia prevention	47 (100%)	126 (100%)	1
No drainage	38 (80.9%)	99 (78.6%)	0.8352
Multimodal analgesia	47 (100%)	126 (100%)	1
No nasogastric tube	46 (97.8%)	119 (94.4%)	0.6847
Early removal of urinary catheter	42 (89.3%)	106 (84.1%)	0.4718
Early mobilization	39 (82.9%)	102 (80.9%)	0.8292
Oral fluids within 6 h from surgery	39 (82.9%)	92 (73%)	0.2319
Opioid-free pain control	4 (89.3%)	109 (86.5%)	0.7987
Prevention of nausea and vomiting	47 (100%)	126 (100%)	1

**Table 5:** Results of primary and secondary outcomes.

Parameters	Group A (Immunonutrition) (*n* = 47)	Group B (Standard) (*n* = 126)	*P*	95% CI
First defecation, days	3.33 ± 2.47	3.96 ± 2.31 (*n* = 125)	0.1193	(−1.42–0.16)
Time of tolerated food intake, days	2.65 ± 1.94	3.54 ± 3.31 (*n* = 125)	0.0848	(−1.9–0.12)
Length hospital stay, days	4.85 ± 2.25	6.06 ± 3.95 (*n* = 125)	0.0492	(−2.44–0.02)
PLOS	5 (10.6%)	25 (20%) (*n* = 125)	0.1814	(0.12–0.22)
Complications	7 (14.9%)	32 (25.4%)	0.1579	(0.16–0.28)
Infective complication	3 (6.3%)	16 (12.6%)	0.2868	(0.07–0.16)
Anastomotic leak	2 (4.3%)	9 (7.1%)	0.7293	(0.03–0.11)
Pneumonia	0 (0%)	3 (2.4%)	0.5636	(0.003–0.004)
Ileus	4 (8.5%)	11 (8.7%)	1	(0.05–0.13)
SSI	1 (2.1%)	10 (7.9%)	0.2928	(0.03–0.11)
Right hemicolectomy	1/16 (6.3%)	7/33 (21.1%)	0.2454	(0.08–0.29)
Left hemicolectomy	0/26 (0%)	1/59 (1.7%)	1	(0.0001–0.07)
Anterior rectal resection	0/4 (0%)	2/28 (7.1%)	1	(0.007–0.21)
Other	0/1 (0%)	0/6 (0%)	1	
Reoperation rate	4 (8.2%)	11 (8.7%)	1	(0.05–0.123)
30 days readmission rate	2 (4.1%)	3 (2.4%)	1	(0.01–0.06)
30 days mortality rate	0 (0%)	1 (0.8%)	1	(0.001–0.03)

## Data Availability

Data supporting reported results can be found in the database of Policlinico Tor Vergata (www.ptvonline.it). Data are protected and access availability must be obtained.
